# The Cholera in Russia

**Published:** 1848-11

**Authors:** 


*The Cholera in Russia.—Riots in consequence of alleged poisoning.*—A letter dated St. Petersburgh, 10th Oct., gives an account of a disturbance which occurred in consequence of the cholera having recommenced its ravages with increased severity, and particularly amongst the poorer classes. A report was circulated amongst the people, that the aristocracy had employed poison to cut them off. They immediately constructed barricades. The troops were about to attack them, when the Emperor arrived on horseback, attended by a single aide-de-camp. The Emperor ordered the troops to fall back, and, ascending the barricade, he addressed the insurgents as follows:

"The cholera, my children, is a chastisement which God inflicts on men, and to which they must submit with resignation. All the reports of poisonings are pure falsehoods, invented by evil-minded persons, who are the enemies of the people."

The insurgents, who had cast themselves on their knees, as in the attitude of prayer, when they perceived their Czar, remained silent, with the exception of two, who commenced a reply. The Czar ordered the insurgents to arrest those two men, and then commanded the troops to return to their barracks. The insurgents immediately seized their comrades and delivered them up to the police. They demolished the barricades and separated peaceably.—*Ibid.*

